# 22q11.2 Deletion Syndrome in Offspring Conceived via Assisted Reproductive Technology Versus Spontaneously

**DOI:** 10.3390/genes17010068

**Published:** 2026-01-06

**Authors:** Jennifer Borowka, Terrence Blaine Crowley, Ashika Mani, Victoria Guinta, Daniel E. McGinn, Bekah Wang, Audrey Green, Lydia Rockart, Oanh Tran, Beverly S. Emanuel, Elaine H. Zackai, Lorraine Dugoff, Kathleen Valverde, Donna M. McDonald-McGinn

**Affiliations:** 1 Master of Science in Genetic Counseling Program, Perelman School of Medicine, University of Pennsylvania, Philadelphia, PA 19104, USA; borowkaj@chop.edu (J.B.);; 2 22q and You Center, Children’s Hospital of Philadelphia, Philadelphia, PA 19104, USA; 3 Division of Human Genetics, Children’s Hospital of Philadelphia, Philadelphia, PA 19104, USA; 4 Department of Pediatrics, Perelman School of Medicine, University of Pennsylvania, Philadelphia, PA 19104, USA; 5 Department of Obstetrics and Gynecology, Perelman School of Medicine, University of Pennsylvania, Philadelphia, PA 19104, USA; 6 Department of Medicine, Perelman School of Medicine, University of Pennsylvania, Philadelphia, PA 19104, USA

**Keywords:** 22q11.2 deletion syndrome, assisted reproductive technology, in vitro fertilization, perinatal outcomes, DiGeorge syndrome, chromosome 22

## Abstract

**Background/Objectives**: The majority of chromosome 22q11.2 deletions are de novo, resulting from meiotic non-allelic homologous recombination (NAHR). While 22q11.2 deletion syndrome (22q11.2DS)-associated phenotypes are well characterized, risk factors leading to NAHR are poorly understood, including the possible relationship with assisted reproductive technology (ART). Here we examined the prevalence of ART conceptions and medical comorbidities in patients with 22q11.2DS vs. spontaneously conceived (SC) patients with 22q11.2DS. **Methods**: Retrospective analysis, under IRB approval, of medical records on 1184 patients with laboratory-confirmed de novo chromosome 22q11.2 deletions was performed. ART conceptions included IVF with and without ICSI. Deletion size and obstetric, family, and medical histories were examined. **Results**: We identified 30 pregnancies conceived using ART (2.57%) compared with the U.S. general population rate of 2.3% (*p*-value = 0.6603). ART and SC sub-cohorts demonstrated no significant differences in deletion size or perinatal outcomes, including preterm birth, multiples, polyhydramnios, or congenital heart disease. Controlling for these factors, neonates conceived via ART were more likely to be admitted to the ICU (aOR = 6.3). **Conclusions**: Pregnancies conceived via ART, and later found to have 22q11.2DS, demonstrated no significant differences in prevalence or perinatal outcomes compared with the U.S. general population. Moreover, NAHR is unrelated to ART in this population. Likewise, associated phenotypic features are unrelated. These data will be reassuring to those families where ART was employed to conceive children who were later found to have 22q11.2DS.

## 1. Introduction

**Figure 1:**
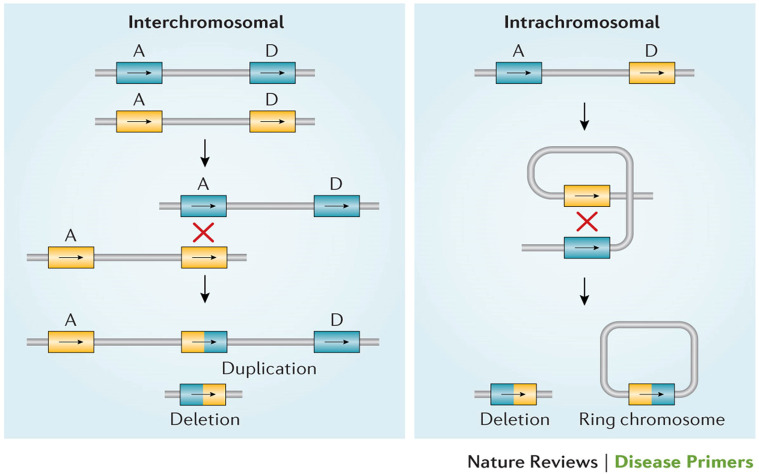
Meiotic non-allelic homologous recombination events demonstrated above. Low copy repeats including A and D, the two most commonly involved in the deletion, are susceptible to interchromosomal and intrachromosomal rearrangements, resulting in either a 22q11.2 deletion or duplication [7].

22q11.2 deletion syndrome (22q11.2DS) is the most common microdeletion syndrome, with an estimated prevalence of 1/2148 live births, 1/1497 miscarriages, and 1/992 pregnancies [1,2,3,4]. Associated features are the result of a hemizygous deletion leading to a broad range of multisystemic problems, including structural anomalies, lifelong medical problems, cognitive deficits, and behavioral phenotypes. These most often include immunodeficiency, congenital heart disease (CHD), palatal abnormalities, endocrine and gastrointestinal differences, developmental delay, and neuropsychiatric illness [5,6,7]. Approximately 90–95% of chromosome 22q11.2 deletions are de novo events [7]. The 22q11.2 deletion, which includes the DiGeorge critical region (DGCR), consists of multiple large regions of duplicated sequences known as low copy repeats (LCRs) or segmental duplications. These nearly identical segmental duplications make the DGCR vulnerable to mispairing during meiotic recombination, resulting in a propensity for non-allelic homologous recombination (NAHR) and leading to the recurrent deletion or duplication [7] (Figure 1).

**Figure 2:**
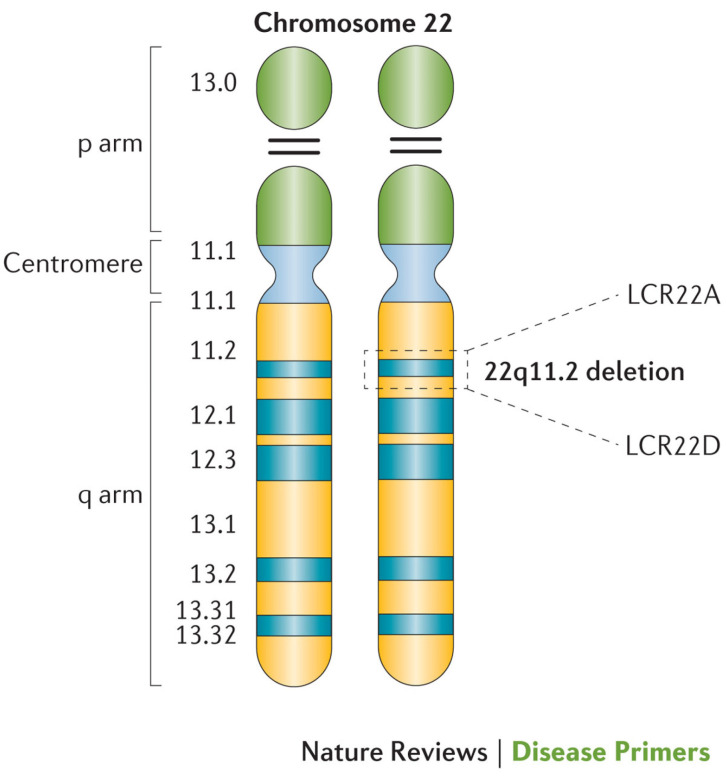
Cytogenetic depiction of chromosome 22. Low copy repeats A and D are highlighted at the 22q11.2 locus, which flanks the typical 3-megabase deletion [7].

**Figure 3:**
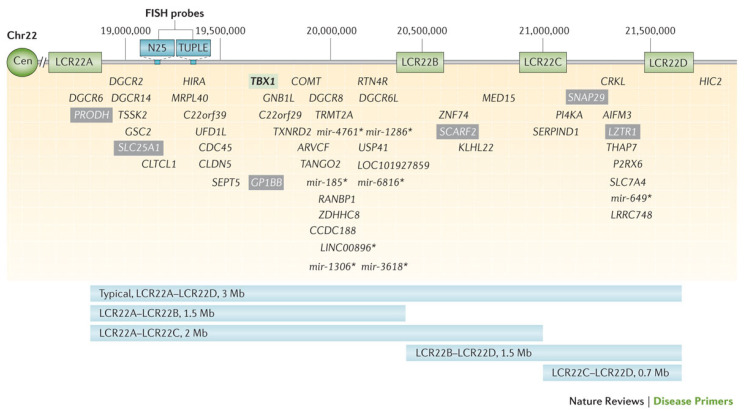
Visual representation of the typical 3-megabase 22q11.2 deletion and genes within the region. Low copy repeats (LCRs) flanking the region (LCR22A, LCR22B, LCR22C, LCR22D) are noted. Select non-coding genes are indicated with an asterisk (*) [7].

The LCRs within the 22q11.2 locus are labeled LCR22A through LCR22H, with the DGCR and most common copy number variants (CNVs) consisting of LCR22A through LCR22D [7,8] (Figure 2). This region includes the critical developmental gene *TBX1*, which plays a vital role in early embryogenesis, including in the formation of the heart, thymus, parathyroid glands, palate, teeth, and craniofacial structures [9]. Likewise, the *CRKL* gene, located between LCR22C and LCR2D, is considered a renal and cardiac developmental driver [10] (Figure 3).

While the phenotypic spectrum of 22q11.2DS has been well characterized in pregnancy, children, and adults, risk factors for occurrence of the microdeletion and the underlying etiology of phenotypic variability are not well understood [5,6,11]. Amongst these risk factors to be explored is the use of assisted reproductive technology (ART).

Approximately 10% of women of reproductive age struggle with infertility [12]. ART has become a valuable tool in reproductive medicine and fertility treatment. Those who employ ART are motivated by the desire to achieve parenthood, to be genetically related to their child, and to experience pregnancy and childbirth [13]. The Centers for Disease Control (CDC) defines ART as all fertility treatments in which either eggs or embryos are handled [14]. These forms of fertility treatment involve the surgical retrieval of eggs from a biologically female person’s body, fertilization by combining egg and sperm in the laboratory, and transfer of the embryo into a uterus. According to the CDC, 2.3% of live births in the United States in 2021 were conceived through ART procedures. Early embryogenesis is very susceptible to environmental conditions. Use of ART embryo culture and exposure to potential differences in temperature, oxygen concentrations, pH, and other unique characteristics of culture media can all adversely affect the embryologic environment [15]. Additionally, the underlying cause of reduced fertility or infertility may contribute to chromosomal errors during the periconceptual period [16].

Although ART is becoming widely used, there may be associated risks that are yet to be well understood. Currently, there is conflicting evidence as to whether ART is associated with an increased risk for copy number variants (CNVs). One report examined neonates conceived through ART who were admitted to intensive care units with suspected genetic diagnoses. No difference in the de novo CNV rate between the ART-conceived and non-ART-conceived sub-cohorts was observed [17]. Another study investigating chromosomal abnormalities following ART, specifically intracytoplasmic sperm injection (ICSI), found 2 cases of de novo 22q11.2DS out of 2505 total pregnancies [18]. While this is a relatively small study, results suggest an increased incidence of the 22q11.2DS microdeletion over the general population rate.

In the current study, we seek to determine the rate of ART conceptions in a cohort of patients with 22q11.2DS and compare this to the general U.S. population rate of ART. Additionally, we aim to examine the possibility that there is a significant difference in perinatal comorbidity in patients with 22q11.2DS conceived via ART versus spontaneously. Moreover, we will examine any potential differences in CNV size in patients with 22q11.2DS conceived via ART versus spontaneously. Our overall aim is to determine if ART predisposes to 22q11.2DS, and, if not, provide reassurance to the families of children conceived via ART who may be attributing the deletion in their offspring to this procedure and potentially blaming themselves for the presence of the condition.

## 2. Materials and Methods

### 2.1. Study Cohort

A retrospective chart review was conducted on 2029 patients enrolled in an IRB-approved study within the 22q and You Center at the Children’s Hospital of Philadelphia (CHOP). All patients had a laboratory-confirmed chromosome 22q11.2 CNV. Two hundred and eighty-five patients with a chromosome 22q11.2 duplication and one hundred and forty-eight familial cases were excluded from this study. Patients with FISH studies only were excluded from the breakpoint sub-analysis, as FISH probes are located between low copy repeats A and B but cannot determine exact breakpoints of the deletion. All patients included in breakpoint analysis had molecular diagnoses via SNP microarray/whole exome sequencing/multiplex ligation probe amplification. Four hundred and ten patients who were enrolled in this study had limited/unknown prenatal and/or birth histories due to adoption, lack of records, or lack of family knowledge of medical history. Sufficient prenatal and birth histories were available for 1186 patients.

ART types included in vitro fertilization (IVF), with and without ICSI. Seventeen patients who were conceived through use of fertility medications and intrauterine insemination (IUI) had data abstracted from the medical records but were excluded from the final analysis based on the CDC’s definition of ART. The final cohort included in the analysis consisted of 1169 patients.

### 2.2. Data Collection

Medical records were reviewed from paper charts and the electronic medical records under an IRB-approved protocol for patients evaluated between 1992 and 2024. Prenatal and birth history data abstraction included use of ART, ART types, pregnancy complications, multiples, gestational age, birth weight, APGAR scores, neonatal intensive care unit (ICU) admission, and presence of congenital heart disease (CHD). Reason for infertility or motivation for ART conception was not available in all cases. Data were entered into a password-protected REDCap version 14 database hosted at CHOP [19,20]. REDCap (Research Electronic Data Capture) is a secure, web-based software platform designed to support data capture for research studies.

### 2.3. Statistical Analysis

Descriptive characteristics were provided for the different variables. The continuous data were expressed as the mean (standard deviation) with medians and range, while the categorical data were summarized by using frequencies and percentages. Demographic and clinical characteristics were compared between participants utilizing ART during pregnancy using a 2-sample *t*-test for continuous variables and a chi-square test for independence for categorical variables.

A 2-sample z-test for proportions to compare ICU admission and preterm status for the ART-conceived group compared to the non-ART-conceived group was performed. A 2-sample *t*-test was computed to compare the difference in means between the ART-conceived group and the non-ART-conceived group for ICU length of stay, mother’s age, father’s age, and age of diagnosis. A chi-square test of independence was used to explore the relationship of ART status with breakpoint size, cardiology findings, and prenatal diagnosis status. A 1-sample z-test for proportions was utilized to examine whether our sample mirrored the actual proportion of those conceived through ART (2.3%).

A multivariate linear regression was performed to measure the contribution of ART status for preterm vs. total birth weight. A multivariate logistic regression was used to examine the potential relationship between ART, polyhydramnios, multiples, and congenital heart disease (CHD).

Statistical tests were conducted at the level α = 0.05. All analyses were completed using R, version 4.

## 3. Results

In total, 30 individuals conceived through ART were identified in our final cohort of 1169 patients with 22q11.2DS (2.57%). Twenty-nine underwent IVF without ICSI, two of whom had general prenatal genetic testing prior to implantation, and one of whom employed a donor egg. One patient underwent IVF with ICSI procedures (Table 1).

We found that the prevalence of ART conceptions in our cohort is not significantly different than the general U.S. population ART rate of 2.3%, as reported by the CDC (*p*-value = 0.6603). Likewise, no significant difference in deletion size was found between the ART and SC groups (*p*-value = 0.4804) (Table 2). Patients whose deletion was identified through FISH studies and, therefore, had unknown breakpoints were excluded from this portion of the analysis.

The age of diagnosis was recorded for 1113 individuals. The average age of diagnosis for the ART group was younger than the average age of diagnosis for the SC group, with a mean age of diagnosis of 1.59 years for the ART group and 2.58 years for the SC group (*p*-value = 0.0259) (Table 3). There was no difference, however, in the rate of prenatal diagnosis between the ART and SC groups (*p*-value = 0.3393).

Maternal age at birth was recorded for 533 individuals and paternal age at birth was known for 457 individuals. Both maternal and paternal ages at time of birth were older in the ART group than the spontaneously conceived (SC) group. Mean maternal age for ART and SC offspring was 35.17 years and 30.18 years, respectively (*p*-value = <0.001), while the mean paternal age for ART offspring and SC offspring was 36.78 years and 32.60 years, respectively (*p*-value = 0.00469) (Table 4).

The mean gestational age was recorded for 495 individuals. Offspring conceived through ART and SC had a mean gestational age of 35.8 weeks and 38.1 weeks, respectively (*p*-value = 0.00137). Multiple gestation pregnancies were more likely to be delivered preterm (<37 weeks) than singleton pregnancies. However, when controlling for the presence of polyhydramnios, singleton vs. multiple gestation, and presence of CHD, there was no significant difference between the gestational term of the ART and SC groups (*p*-value = 0.83018) (Table 5).

Birth weight was recorded for 501 individuals. Mean ART birth weight was lower than in SC individuals, with mean weights of 5.24 lbs and 6.55 lbs, respectively. The mean birth weight of offspring conceived through ART was not significantly different than the mean birth weight for the SC group when adjusting for the presence of polyhydramnios, gestational term, and singleton vs. multiple gestation (*p*-value = 0.1809) (Table 6).

Patients conceived via ART were more likely to be admitted to the intensive care unit (ICU) during the neonatal period than the SC group (adjusted odds ratio = 6.74), even when controlling for polyhydramnios, singleton vs. multiple gestation, and CHD. Of the ART-conceived patients, 70.0% were admitted to the ICU compared to 25.9% of the SC sub-cohort (*p*-value = <0.001) (Table 7). Patients with CHD were more likely to be admitted to the ICU than those without (adjusted odds ratio = 0.56). ICU admission length did not differ statistically between the ART and SC groups (*p*-value = 0.250). The mean lengths of stay for the ART and SC groups were 6.64 weeks and 4.30 weeks, respectively.

CHD data were available for 976 individuals. In total, 14 of 25 patients in the ART group with available cardiac data had CHD (56%). Of the 951 SC individuals with cardiac data, 647 had congenital heart disease (68%) (Table 8). The chi-square test showed no statistical difference between ART and congenital heart disease status (*p*-value = 0.292) in these groups.

## 4. Discussion

In summary, we found no significant difference in the prevalence of 22q11.2DS in patients conceived via ART versus those conceived spontaneously (2.57% in ART-conceived patients compared with 2.3% in the general U.S. population). Additionally, no significant difference in deletion size between the ART and SC groups within our 22q11.2DS population was observed. In fact, even though the overall numbers of patients were small, the rate of nested deletions (LCR22A-LCR2B, LCR22A-LCR22C, LCR22B-LCR22D, and LCR22C-LCR22D) to standard deletions (LCR22A-LCR22D) was essentially the same. Thus, our data suggest that ART is not a risk factor for NAHR or the chromosome 22q11.2 deletion.

For both gestational age and birth weight, the individuals with 22q11.2DS conceived via ART had poorer outcomes than their SC counterparts when comparing the mean values, likely due to multiples resulting in preterm birth. However, when adjusting for potential confounding factors, there were no significant differences between the two groups. This suggests that ART itself is not a driving factor in these outcomes, but impacts the prenatal environment in ways that result in these differences. There is conflicting evidence in the literature as to whether ART is associated with low birth weight and preterm delivery. Many studies have shown that there is a higher rate of low birth weight and preterm delivery in pregnancies conceived via IVF as compared to the general population [17,21]; however, the causes of these outcomes remain poorly understood. Sub-fertility is often cited as a potential cause of unfavorable outcomes in pregnancies conceived via ART, although a 2013 study has shown that even in a mother who has conceived both spontaneously and via ART, the ART offspring are more likely to be born preterm and have a low birth weight. Other potential causes of unfavorable outcomes include factors related to ART techniques such as hormonal stimulation, cryopreservation, culture time, and endometrial conditions [22]. These potential confounders remain to be explored.

Additional studies have shown no significant difference in birth weight between ART and SC cohorts. In a 2019 study comparing perinatal outcomes of 71 patients conceived via ART to 640 patients conceived spontaneously, no significant differences were observed in the birth weight or gestational age between the two groups [23]. Moreover, a 2021 paper examined preterm births in ART vs. SC pregnancies. The results revealed a modest, but not statistically significant, increase in preterm births between the ART and SC groups [24]. In conclusion, our results continue to warrant further exploration of factors contributing to birth weight and gestational age in ART-conceived pregnancies.

In this study, there was a significantly increased risk for ICU admission during the neonatal period between the ART and SC groups, with the ART group being 6.74 times more likely to be admitted to the ICU than SC counterparts. This may be due to many factors s that require further investigation. Patients conceived via ART generally undergo more serial ultrasounds than is typically recommended for a low risk, naturally conceived pregnancy. This increased monitoring and medical attention may result in closer post-natal follow-up, resulting in more/longer admissions. Additionally, other factors such as an increase in multiple gestations leading to prematurity may confound these results. Individuals with CHD were 0.57 times more likely to be admitted to the ICU than those without. Interestingly, there was no statistical difference in the ratio of patients with 22q11.2DS with and without CHD between the ART and SC groups. In the general population, it has been shown that there is a higher rate of CHD in infants conceived via ART [25]. One reason that the increased risk of CHD in our ART sub-cohort may not be observed is due to the increased rate of CHD in all patients with 22q11.2DS (64%) [11]. Since all patients in our cohort are more likely to have a CHD, the additional increased risk in the ART sub-cohort may not be sufficient to confer an increased overall risk. Thus, the finding of an elevated rate of ICU admission for the ART-conceived group versus the SC group requires further investigation.

Notably, patients conceived through ART were more likely to be diagnosed at a younger age than those spontaneously conceived. This difference may be due to greater surveillance of ART pregnancies, or due to higher socio-economic status—as evidenced by parental age—and therefore a greater likelihood to seek diagnostic investigations. In general, those who utilize ART are more likely to be of a higher socio-economic status, allowing more access to medical care and genetic testing. One may argue that an earlier average age of diagnosis in the ART sub-cohort makes ART a protective factor in the long-term outcomes of children with 22q11.2DS.

Timely diagnosis and treatment of 22q11.2DS is essential [3,20]. Approximately 60% of children with 22q11.2DS have hypocalcemia. Undetected and untreated hypocalcemia can result in neonatal seizures or later onset hypocalcemic seizures during times of biologic stress such as during illness, perioperatively, during adolescence, and in pregnancy in women. Hypocalcemic seizures may be a presenting symptom of 22q11.2DS. Timely diagnosis of 22q11.2DS is, therefore, important for endocrine management and prevention of seizures [6,26]. As such, the earlier age at diagnosis in the ART sub-cohort may result in earlier management and protection against comorbid manifestations of the condition.

Similarly, thymic hypoplasia, observed in >75% of patients with 22q11.2DS, requires immunologic evaluation and management as early as possible given the clinical practice recommendations state that immune status should be evaluated prior to administration of live viral vaccines [6]. Again, this supports that early diagnosis of 22q11.2DS is critical for the evaluation and management of all children, particularly in neonates. The ART sub-cohort is, therefore, more likely to be offered comprehensive evaluation and management at a younger age and obviate potentially poorer outcomes. A next step in analysis of this data should include a review of long-term outcomes of children with 22q11.2DS conceived via ART versus spontaneously.

In summary, our study found that although there is a higher rate of early gestational age, low birth weight, and ICU admissions in patients with 22q11.2DS conceived via ART in comparison to spontaneously conceived children with 22q11.2DS, the use of ART is not a risk factor for the deletion itself. Additionally, low birth weight and early gestational age may be influenced by additional prenatal factors such as presence of polyhydramnios, multiples, or the presence of CHD. These factors may be more direct contributors to outcomes rather than ART itself. Our results provide important data on which to base genetic counseling for families whose children with 22q11.2DS were conceived via ART. It also allows providers to reassure families that the occurrence of the chromosome 22q11.2 deletion is not thought to be due to ART and remains a spontaneous event due to the presence of low copy repeats within the DGCR. Families undergoing ART can also be counseled that there is no increased risk for 22q11.2DS when learning about the risks of the technology, although they may be cautioned that preterm birth, lower birth weight, and a greater chance of ICU admission has been observed, given that a causal relationship has not been clearly established.

### 4.1. Limitations

The limitations in this study include the fact that patients were enrolled over a long period of time: born between 1957 and 2024 and enrolled in this study between 1992 and 2024. This may represent a skewed population, as ART was not commonly employed until the 1980s. Additionally, pregnancy history was collected from the pediatric charts, potentially resulting in underreporting of pregnancy complications. Some charts, particularly those where the child was placed for adoption, were lacking documentation of birth history, limiting the denominator for some sub-analyses. Moreover, some features such as CHD were only included in the analysis if an echocardiogram was performed, and palatal anomalies such as velopharyngeal dysfunction could only be included or excluded in children with a sufficient speech sample. Additionally, the primary indication for ART was unknown. These factors contributed to missing data and heterogeneity of cohort subsets used in the analysis. Missing data may have introduced additional bias and create decreased reliability of statistical analysis as it creates inconsistencies among the sub-cohorts. Likewise, an inherent ascertainment bias may exist for the CHOP cohort, given that 50% of patients on average reside >100 miles from Philadelphia and, therefore, may be of higher socio-economic status; hence, they had the means to travel for coordinated multidisciplinary care and, therefore, had more access to ART. This could result in a higher percentage of the cohort being conceived through ART as compared with 22q11.2DS in the general population. Additionally, this bias may result in more in-depth phenotyping of the patients included in the cohort, as they were more likely to have the socio-economic means to travel to a specialized clinic and access multidisciplinary care, leading to earlier diagnosis.

### 4.2. Future Directions

The results of this study warrant further investigation. In particular, it would be valuable to ascertain the number of offspring reported to have 22q11.2DS in a cohort of IVF conceptions. With the increased use of cell-free DNA testing for microdeletions, the ability to detect 22q11.2DS prenatally has increased, including in miscarriages. It would, therefore, be beneficial to establish the number of pregnancies with 22q11.2DS conceived via ART in order to control for the number of pregnancies not carried to term. This might best be accomplished by way of a collaboration with an NIPT testing company where hundreds of thousands of pregnancies are monitored annually. Moreover, the elevated rate of ICU admission for the ART-conceived group versus the SC group requires further investigation, including examining this rate in patients conceived via ART without 22q11.2DS.

## Figures and Tables

**Table 1:** Method of ART conception.

IVF (n)	PGD (General Screen) (n)	Donor Egg (N)	ICSI (n)
24	2	3	1

**Table 2:** 22q11.2 deletion breakpoints.

LCR Breakpoints	ART (n)	SC (n)
A-B	1	41
A-C	1	16
A-D	24	735
B-D	0	10
C-D	1	5
Distal	0	22
Non-LCR-Mediated	0	10

**Table 3:** Mean age at diagnosis.

ART (Years)	SC (Years)	*p*-Value
1.59	2.58	0.0259

**Table 4:** Mean parental ages.

	ART (Years)	SC (Years)	*p*-Value
Maternal Age (n = 533)	35.17	30.18	<0.01
Paternal Age (n = 457)	36.78	32.60	0.00469

**Table 5:** Mean gestational age.

ART (Weeks)	SC (Weeks)	*p*-Value	*p*-Value (Adjusted for Polyhydramnios, Multiple Gestation, Presence of CHD)
35.8	38.1	0.00137	0.83018

**Table 6:** Mean birth weight.

ART (lbs)	SC (lbs)	*p*-Value	*p*-Value (Adjusted for Term, Polyhydramnios, Multiple Gestation, Presence of CHD)
5.24	6.55	<0.001	0.1809

**Table 7:** Rate of ICU admission.

ART (n)	SC (n)	*p*-Value	*p*-Value (Adjusted for Term, Polyhydramnios, Multiple Gestation, Presence of CHD)
21 (70%)	294 (25.9%)	<0.001	<0.001

**Table 8:** Congenital heart differences.

	ART (n)	SC (n)
CHD Present	14	647
CHD Absent	11	304

## Data Availability

Any data requests can be directed to the corresponding author.
